# Addressing Efficacy Uncertainties in Expedited Programs for Gene Therapy Products: A Comparative Assessment of Post-Approval Requirements by the FDA and EMA

**DOI:** 10.3390/biotech15040082

**Published:** 2026-09-25

**Authors:** Kimika Nakamura, Yasuyuki Takashima, Shinya Watanabe, Aiki Marushima, Koichi Hashimoto

**Affiliations:** 1 Department of Clinical and Translational Research Methodology, Institute of Medicine, University of Tsukuba, 1-1-1 Tennodai, Tsukuba 305-8575, Ibaraki, Japan; 2 Doctoral Program in Medical Sciences, Graduate School of Comprehensive Human Sciences, University of Tsukuba, 1-1-1 Tennodai, Tsukuba 305-8575, Ibaraki, Japan; 3 Tsukuba Clinical Research & Development Organization, University of Tsukuba, 2-1-1 Amakubo, Tsukuba 305-8576, Ibaraki, Japan; ytakashima@md.tsukuba.ac.jp (Y.T.); aiki.marushima@md.tsukuba.ac.jp (A.M.); 4 Department of Neurosurgery, Mito Kyodo General Hospital, Tsukuba University Hospital Mito Area Medical Education Center, 3-2-7 Miyamachi, Mito 310-0015, Ibaraki, Japan; shinya-watanabey@md.tsukuba.ac.jp; 5 Department of Neurosurgery, Institute of Medicine, University of Tsukuba, 1-1-1 Tennodai, Tsukuba 305-8575, Ibaraki, Japan; 6 Center for Cybernics Research, University of Tsukuba, 1-1-1 Tennodai, Tsukuba 305-8575, Ibaraki, Japan

**Keywords:** gene therapy, expedited program, accelerated approval, conditional marketing authorization, post-approval requirements

## Abstract

Expedited programs accelerate the availability of gene therapy products but frequently leave uncertainties regarding efficacy at approval. How post-approval requirements are utilized to complement these specific evidence gaps remains unclear. To address this, we conducted a detailed analysis of 18 gene therapy products granted Accelerated Approval (AA) by the FDA or conditional marketing authorization (CMA) by the EMA between January 2015 and September 2025. By reviewing official regulatory documents, we examined the efficacy-related uncertainties at the time of approval and the corresponding post-approval requirements. For AA, six of eight products (75.0%) were based on surrogate biomarkers or tumor response-based endpoints, with postmarketing requirements aimed at confirmatory evidence to verify clinical benefit. In contrast, seven of eleven CMA products (63.6%) were supported by disease-specific clinical outcomes, with specific obligations requiring final study results, extended follow-up, additional clinical studies, or registry-based data collection to complete the evidence package. Descriptive differences in post-approval evidence requirements were observed among the FDA AA and EMA CMA products included in this study, although the sample was limited. Early engagement with regulatory authorities may support flexible development strategies aligned with evolving regulatory expectations. It may also support post-approval evidence planning to address residual uncertainty.

## 1. Introduction

Gene therapy products are innovative medical technologies aimed at the fundamental treatment of diseases, and they are particularly anticipated in the fields of genetic disorders, cancer and infectious diseases. Gene therapy is designed to achieve therapeutic effects through the expression of genetic material introduced into the body via viral vectors or other methods [1,2]. Gene therapy can be classified into in vivo (direct delivery of genetic material to patients) and ex vivo (re-administration after genetic modification of patients’ cells outside the body) gene therapy [2,3,4]. For these gene therapy products to be made available for clinical use, they must obtain approval from regulatory authorities, as with conventional medical drugs. Regulatory agencies have established multiple review pathways, including expedited programs, to address unmet medical needs for serious or life-threatening diseases [5,6,7]. Expedited programs can help address unmet medical needs and facilitate earlier patient access by balancing risks and benefits, considering uncertainties, and providing regulatory flexibility [6,7,8,9]. In particular, for gene therapy products—where curative therapeutic effects are anticipated, but development hurdles are high—expedited programs play a critical regulatory role by enabling earlier patient access and allowing development to proceed in a stepwise manner while acknowledging the uncertainties inherent in the development process.

In practice, some gene therapy products utilize expedited programs, such as the Food and Drug Administration (FDA)’s accelerated approval (AA) and the European Medicines Agency (EMA)’s conditional marketing authorization (CMA) [5,6,7]. However, these programs face several challenges, including residual uncertainty arising from limited comprehensive clinical evidence at the time of approval [8,9,10], delays or non-implementation of post-approval clinical trials [11,12,13], and uncertainties regarding long-term efficacy and safety [13,14,15].

Although similar expedited programs exist at regulatory authorities outside the FDA and EMA (e.g., Japan’s conditional and time-limited approval program), their utilization has been limited compared with that in the United States and Europe [5]. Because the number of approved gene therapy products in regions outside the United States and Europe remains limited, it is anticipated that more proactive use of expedited programs will help accelerate development and enable patients to gain earlier access to innovative gene therapy products. Therefore, organizing the post-approval requirements related to expedited programs for gene therapy products in the United States and Europe is considered useful for the development of future gene therapy products and for advancing development in other regions. Several studies have reported overviews of expedited programs and development trends for gene therapies [9,16,17]. However, these studies have not thoroughly analyzed the relationship between the specific efficacy uncertainties remaining at the time of expedited approval and the post-approval requirements subsequently imposed to address those uncertainties. It remains unclear how approval-time efficacy uncertainties are addressed through post-approval requirements for gene therapy products approved on limited clinical evidence.

To address this gap, we conducted a detailed analysis of gene therapy products granted either AA by the FDA or CMA by the EMA. This study aims to (1) identify the key efficacy uncertainties present at the time of expedited approval and (2) characterize the corresponding post-approval evidence required by each agency, thereby clarifying the distinct regulatory approaches used to complement the evidence package for these gene therapy products.

## 2. Materials and Methods

### 2.1. Study Selection and Eligibility Criteria

The products were identified using information on approval regions and approval years from the Q3 2025 Gene, Cell, & RNA Therapy Landscape Report [18] and a summary of gene therapy products approved in Japan, the US, and Europe [19]. Eligibility and approval information were subsequently verified using publicly available regulatory documents for each product available on the FDA or EMA websites, including FDA Approval Letters and EMA Medicine Overviews, as well as Clarivate’s Cortellis database. Products approved between 1 January 2015 and 30 September 2025 were included in the study, whereas mRNA-based products were excluded. No products meeting the eligibility criteria were excluded from the study.

We identified gene therapy products that received FDA AA or EMA CMA between 1 January 2015 and 30 September 2025. A total of 18 products were included: 6 in vivo products, 8 ex vivo CAR-T products, and 4 ex vivo other than CAR-T products. Table 1 summarizes the products included in this study and the approval pathway of each product. This study does not include nucleic acid drugs or mRNA drugs. Because one product received both FDA AA and EMA CMA, pathway-specific analyses included eight FDA AA products and eleven EMA CMA products.

### 2.2. Regulatory Data Sources

In this study, we analyzed the post-approval requirements associated with expedited pathways, specifically FDA postmarketing requirements and EMA-specific obligations. To investigate the approval pathways, key efficacy endpoints, and post-approval requirements associated with expedited programs, we evaluated the following public documents and databases:

- FDA sources: Summary Basis for Regulatory Action documents, Approval Letters, product labeling, the FDA Approved Cellular and Gene Therapy Products webpage [20], the Accelerated Approvals Database webpage [21], and the Postmarketing Requirements and Commitments: Searchable Database [22];
- EMA sources: Medicine Overviews and European Public Assessment Reports [23] and official EMA websites for each product.

For FDA-approved products, Approval Letters issued at the time of approval for the relevant indication were reviewed to determine whether AA had been granted and to identify the indications. For EMA-approved products, EMA Medicine Overviews and European Public Assessment Reports were reviewed to determine whether CMA had been granted and to identify the indication. This study focused specifically on efficacy-related uncertainties and the corresponding post-approval evidence requirements. Safety and chemistry, manufacturing and controls (CMC)-related requirements were beyond the scope of the analysis.

### 2.3. Extraction of Key Efficacy Endpoints and Remaining Uncertainties

Key efficacy endpoints at the time of approval were extracted from the Summary Basis for Regulatory Action for FDA AA and from the European Public Assessment Report for EMA CMA.

Remaining uncertainty at approval was defined as the principal unresolved efficacy-related question descriptively characterized from limitations, uncertainties, or evidence gaps in the approval-time efficacy evidence and publicly available FDA and EMA regulatory assessment documents, considered together with the corresponding post-approval evidence requirements. The integrated review included FDA Summary Basis for Regulatory Action documents, such as the Benefit–Risk Assessment section, EMA European Public Assessment Reports, such as the Benefit–Risk Assessment and discussion section, FDA postmarketing requirements, and EMA specific obligations. This review was used to descriptively characterize the regulatory relationship between efficacy uncertainties characterized from the reviewed sources and the evidence expected after approval, rather than to independently validate their correspondence. To facilitate comparison between FDA and EMA products, remaining uncertainties were summarized using a standardized “Whether…” format.

For descriptive analysis, approval-time evidence was classified into two categories: (1) surrogate biomarkers or tumor response-based endpoints and (2) disease-specific clinical outcomes. Tumor response-based endpoints included overall response rate (ORR), duration of response (DOR), and complete remission-based response measures (CR/CRi). For products supported by both biomarker-based measures and direct clinical outcomes, classification was based on the presence of a direct clinical outcome. To further distinguish different types of approval-time evidence, Table 2 and Table 3 additionally indicate whether each endpoint represents a direct clinical outcome, surrogate biomarker, or tumor response-based endpoint. These labels were added to provide additional information on the type of approval-time evidence and did not alter the evidence categories used in the primary analysis.

### 2.4. Definition of Post-Approval Evidence

FDA postmarketing requirements were identified from the “Recommendation for Postmarketing Activities” section of the Summary Basis for Regulatory Action and Postmarketing Requirements and Commitments: Searchable Database for each product. EMA-specific obligations were identified from the “Specific Obligation to complete post-authorization measures for the conditional marketing authorisation” section of the European Public Assessment Report for each product.

To ensure clarity, where multiple FDA postmarketing requirements or EMA-specific obligations were imposed on a single product, the required evidence was reviewed collectively and summarized to represent the overall evidence requested by the respective regulatory authority. For descriptive frequency reporting, product-level post-approval evidence requirements were grouped into four categories: extended follow-up or maturation of outcomes, randomized comparative studies, registry-based evidence collection, and additional cohort or patient population evidence. Because a single product could include multiple types of post-approval evidence requirements, the same product could be counted in more than one category. Each product was counted once within each applicable category.

Notably, post-approval requirements were identified and evaluated at the indication level. For presentation purposes, these indication-specific findings were subsequently summarized at the product level. The analysis was restricted to indications granted expedited approval (AA or CMA); indications receiving standard approval were excluded.

All product selection, data extraction, categorization, and summarization procedures were conducted by the first author (K.N.). The overall findings and their interpretation were reviewed and discussed among the co-authors (S.W. and K.H.).

## 3. Results

The relationship between the remaining efficacy uncertainties at the time of approval and post-approval evidence required is summarized separately for the FDA AA (Table 2) and EMA CMA (Table 3). Detailed source information and post-approval requirement descriptions derived from the respective regulatory documents are provided in Table S1 (FDA AA) and Table S2 (EMA CMA). The frequency of post-approval evidence requirement categories among gene therapy products granted FDA AA or EMA CMA is shown in Table S3.

### 3.1. FDA Accelerated Approval

Among the eight products granted AA by the FDA, six (75.0%) were approved based on surrogate biomarkers or tumor response-based endpoints, including micro-dystrophin expression for delandistrogene moxeparvovec and tumor response-based measures for five oncology products, whereas two products (25%) were approved based on a disease-specific clinical outcome. The corresponding postmarketing requirements primarily aimed to provide confirmatory evidence to verify the clinical benefit beyond the approval-time evidence.

The corresponding postmarketing requirements included additional clinical endpoints, mature outcome data, or extended follow-up. For oncology products initially approved based on ORR, the postmarketing requirements called for confirmation of clinical benefit through mature response data, extended follow-up, additional cohorts, or more definitive outcomes such as progression-free survival. The postmarketing requirements varied across products and included randomized comparative studies, extended follow-up studies and additional cohort evaluations.

Outside of oncology, delandistrogene moxeparvovec, which was approved based on a surrogate biomarker (micro-dystrophin expression), received a postmarketing requirement to assess clinical motor function. Additionally, for elivaldogene autotemcel, the postmarketing requirements mandated long-term evaluation of efficacy and safety, including assessment of long-term neurological outcomes and survival.

### 3.2. EMA Conditional Marketing Authorisation

Among the eleven products granted CMA by the EMA, seven (63.6%) were supported by disease-specific clinical outcomes and four (36.4%) by tumor response-based endpoints. Disease-specific clinical outcomes included bleeding-related outcomes in hemophilia, event-free survival and motor milestone achievement in spinal muscular atrophy, overall survival in the adjunctive treatment setting of haploidentical Hematopoietic Stem Cell Transplantation, and transfusion independence or vaso-occlusive crisis-related outcomes in hemoglobinopathies.

The corresponding specific obligations included the final study results, additional clinical studies, extended follow-up, and registry-based evidence collection. For example, for the hemophilia treatments fidanacogene elaparvovec, etranacogene dezaparvovec, and valoctocogene roxaparvovec, the specific obligations primarily focused on long-term evaluation through extended follow-up. For several oncology products, specific obligations required additional confirmation through randomized comparative studies, prospective registry-based studies, or registry-based data collection.

A descriptive frequency summary of product-level post-approval evidence requirement categories is provided in Table S3. Products could contribute to more than one category when multiple types of post-approval evidence requirements were assigned.

## 4. Discussion

This study suggests that, for expedited programs of gene therapy products by the FDA and EMA, the efficacy evidence available at the time of approval, although often limited and associated with uncertainty, was accompanied by post-approval requirements that appeared to be associated with specific clinical uncertainties present at the time of approval. The nature of the requirements appeared to vary according to the approval-time evidence, the completeness of the available clinical data and product characteristics.

As shown in Table 4, previous studies have reported an overview of expedited programs at each regulatory authority and the development trends of gene therapy [5,9,16,17,24,25,26]. However, previous studies have not thoroughly examined the relationship between approval-time evidence, remaining efficacy uncertainties, and the corresponding post-approval evidence requirements for gene therapy products approved through expedited pathways. This study addresses this gap by analyzing post-approval requirements in detail and clarifying how they are utilized to complement the specific clinical uncertainties remaining at the time of expedited approval.

### 4.1. Differences in Regulatory Frameworks Between FDA AA and EMA CMA

An important observation of this study was that different patterns of post-approval requirements were identified among the products granted FDA AA and EMA CMA. These patterns may partly reflect differences in the regulatory frameworks for expedited pathways, as well as differences in the products and indications included in each pathway. Among FDA AA products, 5 of 8 products were approved for oncology indications, whereas among EMA CMA products, 4 of 11 products were approved for oncology indications and the remainder were approved for non-oncology indications. Therefore, differences in the distribution of indication types may have contributed to the observed differences in efficacy endpoints and post-approval requirements.

AA may be granted based on a surrogate endpoint or an intermediate clinical endpoint that is reasonably likely to predict clinical benefit, thereby allowing for earlier approval for drugs that treat serious conditions and address an unmet medical need [21]. Consequently, the primary uncertainty at approval is whether the surrogate biomarker or tumor response-based endpoint predicts or translates into clinical benefit. Post-approval requirements for products approved based on tumor response-based endpoints such as ORR required additional evidence, including mature response data, extended follow-up, additional cohorts, or more definitive outcomes such as progression-free survival.

Furthermore, the design of postmarketing studies varied according to the nature of the remaining uncertainty. While extended follow-up played an important role in assessing durability or long-term outcomes for some products (e.g., brexucabtagene autoleucel), others required randomized comparative studies or additional studies to further confirm clinical benefit (e.g., axicabtagene ciloleucel).

Conversely, CMA does not include a direct equivalent to the AA surrogate endpoint. Instead, CMA is granted based on the following criteria: the medicine must address an unmet medical need, show a positive benefit–risk balance, and offer immediate patient benefits that outweigh the risks of incomplete data. A CMA requires that the applicant is likely to provide comprehensive clinical data post-authorization [7]. Because CMA is granted despite remaining limitations in the available evidence, specific obligations identified in our study may serve to complement the available evidence after approval. For gene therapies targeting chronic conditions such as hemophilia (e.g., fidanacogene elaparvovec), specific obligations often included extended follow-up as part of a broader strategy to generate more comprehensive efficacy and safety evidence. In other cases, obligations included submission of the final study results, additional clinical studies, or registry-based evidence collection to further characterize efficacy and safety. Among CAR-T products granted EMA CMA, the additional evidence requirements varied. Registry-based studies were required for some products, such as obecabtagene autoleucel, whereas randomized comparative studies were required for others, including ciltacabtagene autoleucel and idecabtagene vicleucel.

These differing post-approval requirements are illustrated by brexucabtagene autoleucel, the only product in this study granted both FDA AA and EMA CMA. While both regulators required extended follow-up of the pivotal ZUMA-2 trial to assess the durability of response, their additional requirements differed. The FDA postmarketing requirement included an additional cohort of BTK inhibitor-naïve patients to further evaluate efficacy in a population not represented in the approval-time evidence. In contrast, the EMA specific obligations included prospective registry-based studies to collect additional efficacy and safety data in routine clinical practice. This example might illustrate the different post-approval approaches applied to the products evaluated by each regulator.

In summary, both FDA AA and EMA CMA rely on post-approval evidence generation to address efficacy uncertainties remaining at the time of approval. Our findings suggest that FDA postmarketing requirements frequently aim to confirm clinical benefit following approvals based on surrogate biomarkers or tumor response-based endpoints, whereas EMA specific obligations more often focus on completing and maturing the evidence package through extended follow-up, final study results, and registry-based data collection.

### 4.2. Practical Considerations for Gene Therapy Development

The findings of this study may provide practical considerations for biotechnology companies developing gene therapies. An important practical consideration is that post-approval requirements (AA; postmarketing requirements and CMA; specific obligations) are an essential part of the evidence generation lifecycle.

Consideration of FDA and EMA guidance may support post-approval evidence planning. Recent FDA draft guidance documents address innovative clinical trial designs for small populations [27] and oncology therapeutics [28]. In addition, for developers pursuing EMA CMA, specific obligations may have important implications for post-approval evidence generation and data collection in accordance with relevant EMA guidance and CMA implementation guidelines [29,30].

In addition to regulatory considerations, practical challenges associated with post-approval evidence generation should also be acknowledged for gene therapy products. Many gene therapies target rare diseases and are developed in small patient populations, while long-term follow-up is often required to evaluate the durability of treatment effects. These characteristics may complicate the timely completion of post-approval studies. Challenges associated with accelerated approval of gene therapies have also been discussed previously [13]. Recent FDA draft guidance documents provide recommendations on trial planning and innovative study designs for cell and gene therapies developed in small populations [27] and on trial design to support accelerated approval of oncology therapeutics [28]. In addition, recent FDA draft guidance has emphasized that confirmatory studies should be underway before approval or within a specified period after approval to facilitate the timely completion of post-approval studies [31]. Such recommendations may contribute to the timely completion of post-approval studies and help reduce delays in confirmatory evidence generation.

These findings may provide useful context for post-approval planning in gene therapy development under expedited pathways. Early engagement during regulatory consultations may help developers to align with these evolving expectations, thereby supporting more efficient development and subsequent evidence collection.

### 4.3. Study Limitations

This study has several limitations. First, the products investigated in this study are limited to those approved by the FDA AA or EMA CMA as of 30 September 2025, excluding products under development or granted regular approval or approved by other regulatory authorities. Second, this analysis focused on efficacy-related post-approval requirements associated with expedited programs (AA; postmarketing requirements, CMA; specific obligations). Therefore, the postmarketing requirements for products (e.g., postmarketing surveys, safety monitoring and CMC) have not been investigated. Third, while safety concerns are discussed as part of the regulatory context, this study did not systematically assess post-approval safety outcomes, including safety signals emerging after approval. Fourth, while post-approval requirements were identified and evaluated at the indication level, findings were consolidated at the product level for presentation. As a result, indication-specific differences may not be fully reflected for products with multiple expedited approval indications. Differences observed between FDA AA and EMA CMA may partly reflect differences in the products and indications included in this analysis, rather than regulatory framework characteristics alone. For products with multiple expedited indications, including delandistrogene moxeparvovec, lisocabtagene maraleucel, and brexucabtagene autoleucel, indication-specific differences were observed in patient populations, study designs, and post-approval evidence requirements. However, these differences remained within the same higher-level descriptions of the approval-time evidence, remaining uncertainties, and post-approval evidence requirements used in this study. Accordingly, product-level aggregation is unlikely to have materially influenced the overall interpretation of the findings.

Finally, while this study highlights different approaches to post-approval evidence generation under FDA AA and EMA CMA, only one product (brexucabtagene autoleucel) had been granted both expedited pathways. In addition, the number of products included in this study was limited, and no statistical comparisons were performed. Consequently, direct comparison between FDA AA and EMA CMA products was limited, and the findings should be interpreted as descriptive findings from the products included in this study rather than definitive differences between the two frameworks. Additionally, differences in the distribution of indication types between FDA AA and EMA CMA products may have influenced the observed patterns of efficacy endpoints and post-approval requirements.

Product selection, data extraction, and categorization of efficacy endpoints, remaining uncertainties, and post-approval requirements were conducted by a single reviewer. The overall findings and their interpretation were reviewed and discussed among co-authors.

The relationship between remaining uncertainties and post-approval evidence was assessed descriptively. Because post-approval evidence requirements were also considered when characterizing remaining uncertainties, the characterization of remaining uncertainties and the assessment of their relationship with post-approval evidence were partially dependent. Accordingly, this analysis was not intended to independently validate correspondence between remaining uncertainties and post-approval evidence. Therefore, the findings should be interpreted as a descriptive evaluation rather than an independent validation of correspondence between remaining uncertainties and post-approval evidence.

Despite these limitations, by focusing on gene therapy products approved under expedited programs by the FDA and EMA, this study offers a detailed assessment of regulatory practices in this area. The findings in this study may help future regulatory discussions on how to integrate evidence available at the time of approval with data generated post-approval.

## 5. Conclusions

This study provides a descriptive assessment of approval-time efficacy uncertainties and the corresponding post-approval evidence requirements among gene therapies granted FDA AA or EMA CMA. Among the FDA AA products included in this study, six of eight products (75.0%) were approved based on surrogate biomarkers or tumor response-based endpoints, and the corresponding postmarketing requirements frequently involved evidence intended to confirm clinical benefit. In contrast, among the EMA CMA products included in this study, seven of eleven products (63.6%) were supported by disease-specific clinical outcomes, and the corresponding EMA-specific obligations often included extended follow-up, final study results, and registry-based data collection to further develop the available evidence. These findings should be interpreted as descriptive observations based on the products included in this study rather than evidence of definitive differences between regulatory frameworks.

To support expedited development and early patient access, it may be useful to consider clinical trial design, endpoint selection, and post-approval data generation from the early stages of development. As a practical consideration, developers may benefit from early and continuous communication with regulatory authorities while remaining responsive to evolving regulatory trends.

## Figures and Tables

**Table 1:** Gene Therapy Products Included in the Analysis and Their FDA and EMA Approval Pathways.

Modality	Pharmaceutical Name	Product Name	FDA Pathway	EMA Pathway
In vivo	fidanacogene elaparvovec ^a^	BEQVEZ^®^	Traditional approval	CMA
In vivo	eladocagene exuparvovec	KEBILIDI^®^ /UPSTAZA^®^	AA	Standard marketing authorization
In vivo	delandistrogene moxeparvovec	ELEVIDYS^®^	AA and traditional approval ^b^	Not approved
In vivo	etranacogene dezaparvovec	HEMGENIX^®^	Traditional approval	CMA
In vivo	valoctocogene roxaparvovec	ROCTAVIAN^®^	Traditional approval	CMA
In vivo	onasemnogene abeparvovec	ZOLGENSMA^®^	Traditional approval	CMA (later transitioned to standard marketing authorization)
Ex vivo CAR-T	obecabtagene autoleucel	AUCATZYL^®^	Traditional approval	CMA
Ex vivo CAR-T	afamitresgene autoleucel	TECELRA^®^	AA	Not approved
Ex vivo CAR-T	lisocabtagene maraleucel ^c^	BREYANZI^®^	AA	Standard marketing authorization
Ex vivo CAR-T	ciltacabtagene autoleucel	CARVYKTI^®^	Traditional approval	CMA (later transitioned to standard marketing authorization)
Ex vivo CAR-T	tisagenlecleucel ^c^	KYMRIAH^®^	AA	Standard marketing authorization
Ex vivo CAR-T	idecabtagene vicleucel	ABECMA^®^	Traditional approval	CMA (later transitioned to standard marketing authorization)
Ex vivo CAR-T	axicabtagene ciloleucel ^c^	YESCARTA^®^	AA	Standard marketing authorization
Ex vivo CAR-T	brexucabtagene autoleucel ^c^	TECARTUS^®^	AA	CMA
Ex vivo other than CAR-T	exagamglogene autotemcel	CASGEVY^®^	Traditional approval	CMA
Ex vivo other than CAR-T	elivaldogene autotemcel ^a^	SKYSONA^®^	AA	Standard marketing authorization
Ex vivo other than CAR-T	betibeglogene autotemcel ^a^	ZYNTEGLO^®^	Traditional approval	CMA
Ex vivo other than CAR-T	nalotimagene carmaleucel ^a^	ZALMOXIS	Not approved	CMA

Products were included if at least one indication had received FDA AA or EMA CMA during the study period. The pathway shown for the other authority may therefore be traditional approval, standard marketing authorization, or not approved. a. Marketing authorization in the European Union was subsequently withdrawn at the request of the marketing authorization holder. b. Delandistrogene moxeparvovec was approved for ambulatory and non-ambulatory patients with DMD. In ambulatory patients aged ≥ 4 years, delandistrogene moxeparvovec transitioned from accelerated to regular approval, while accelerated approval was granted for non-ambulatory patients aged ≥ 4 years in 2024. c. For lisocabtagene maraleucel, brexucabtagene autoleucel, axicabtagene ciloleucel, and tisagenlecleucel, expedited regulatory pathways were granted only for specific indications (accelerated approval for FL and/or CLL/SLL for lisocabtagene maraleucel, MCL for brexucabtagene autoleucel, and FL for axicabtagene ciloleucel and tisagenlecleucel). These products also have other indications approved through standard regulatory pathways. Analyses of approval-time evidence and postmarketing requirements were restricted to the indications granted expedited approval pathways. AA = accelerated approval; CMA = conditional marketing authorization; DMD = Duchenne muscular dystrophy; CLL = Chronic lymphocytic leukemia; FL = Follicular lymphoma; MCL = mantle cell lymphoma; SLL = small lymphocytic lymphoma.

**Table 2:** Approval-time evidence, remaining uncertainty at approval, and post-approval evidence requirements for gene therapies granted FDA Accelerated Approval.

Pharmaceutical Name	Indication	Approval-Time Evidence	Remaining Uncertainty at Approval	Post-Approval Evidence Required (FDA Postmarketing Requirements)
eladocagene exuparvovec	AADC deficiency	Gross motor function improvement ^a^	Whether early improvements in motor function translate into broader clinical benefit across major clinical features of AADC deficiency.	Submission of clinical study reports and datasets evaluating broader treatment effects across major manifestations of AADC deficiency.
delandistrogene moxeparvovec	DMD (ambulatory or non-ambulatory patients)	Micro-dystrophin expression ^b^	Whether micro-dystrophin expression translates into clinically meaningful benefit across ambulatory and non-ambulatory patients with DMD.	Confirmation of clinical benefit through randomized comparative studies in ambulatory and non-ambulatory DMD populations.
afamitresgene autoleucel	Synovial sarcoma	ORR, supported by DOR ^c^	Whether the observed response rate and duration of response translate into durable and clinically meaningful benefit.	Submission of final clinical study report with more precise estimation of ORR and mature DOR.
lisocabtagene maraleucel	FL, CLL/SLL	ORR, supported by DOR ^c^	Whether observed responses translate into durable clinical benefit across approved hematologic malignancy populations.	Long-term evaluation of the durability of response, with additional confirmation in broader hematologic malignancy populations.
tisagenlecleucel	FL	ORR, supported by DOR ^c^	Whether response-based evidence translates into improved progression-free survival compared with standard therapy.	Confirmation of efficacy through a randomized comparative study in FL.
axicabtagene ciloleucel	FL	ORR, supported by DOR ^c^	Whether response-based evidence translates into improved progression-free survival compared with standard therapy.	Confirmation of efficacy through a randomized comparative study in FL.
brexucabtagene autoleucel	MCL	ORR, supported by DOR ^c^	Whether observed responses are maintained over time and can be confirmed in MCL populations.	Long-term evaluation of efficacy, with additional evidence in BTK inhibitor-naïve MCL populations.
elivaldogene autotemcel	CALD	MFD-free survival ^a^	Whether event-free survival without major functional disability can be reproduced in additional patients and maintained over the long term.	Long-term evaluation of efficacy, including assessment of event-free survival and long-term outcomes.

Remaining uncertainty at approval was descriptively characterized through an integrated review of approval-time efficacy evidence, FDA regulatory assessment documents, and the postmarketing requirements. The resulting summary describes the regulatory relationship between the unresolved efficacy question and the evidence expected after approval. Postmarketing evidence required was summarized from postmarketing requirements issued at the time of accelerated approval. a. Approval-time evidence representing a direct clinical outcome. b. Approval-time evidence representing a surrogate biomarker. c. Approval-time evidence representing a tumor response-based endpoint, including ORR, DOR, and CR/CRi. AADC = aromatic L-amino acid decarboxylase; BTK = Bruton’s tyrosine kinase; CALD = cerebral adrenoleukodystrophy; CLL = chronic lymphocytic leukemia; DMD = Duchenne muscular dystrophy; DOR = duration of response; FL = follicular lymphoma; MCL = mantle cell lymphoma; MFD = major functional disability; ORR = overall response rate; SLL = small lymphocytic lymphoma.

**Table 3:** Approval-time evidence, remaining uncertainty at approval, and post-approval evidence requirements for gene therapies granted EMA conditional marketing authorization.

Pharmaceutical Name	Indication	Approval-Time Evidence	Remaining Uncertainty at Approval	Post-Approval Evidence Required (EMA Specific Obligations)
fidanacogene elaparvovec	Hemophilia B	Annualized bleeding rate and factor IX activity ^a^	Whether reductions in bleeding and factor IX activity are maintained over the long term.	Long-term evaluation of efficacy and safety through extended follow-up of clinical studies.
etranacogene dezaparvovec	Hemophilia B	Annualized bleeding rate and factor IX activity ^a^	Whether reductions in bleeding and factor IX activity are maintained over the long term.	Long-term evaluation of the durability of efficacy and safety through extended follow-up of clinical studies.
valoctocogene roxaparvovec	Hemophilia A	Annualized bleeding rate and FVIII activity ^a^	Whether factor VIII activity and bleeding reduction are maintained over the long term.	Long-term evaluation of the durability of efficacy and safety, with additional evidence on corticosteroid regimens.
onasemnogene abeparvovec	SMA	Event-free survival and motor milestone achievement ^a^	Whether survival and motor milestone benefits after treatment are maintained over the long term.	Long-term evaluation of efficacy, safety, and tolerability following treatment in symptomatic and presymptomatic SMA populations.
obecabtagene autoleucel	ALL	Overall remission rate (CR/CRi) ^b^	Whether initial remission responses translate into durable clinical benefit and are confirmed in clinical practice.	Long-term evaluation of efficacy and safety, with additional confirmation through a prospective registry study.
ciltacabtagene autoleucel	Multiple myeloma	ORR and DOR ^b^	Whether observed responses translate into durable clinical benefit.	Long-term evaluation of efficacy and safety, with additional confirmation through a randomized comparative study.
idecabtagene vicleucel	Multiple myeloma	ORR and DOR ^b^	Whether observed responses translate into durable clinical benefit.	Long-term evaluation of efficacy and safety, with additional confirmation through extended follow-up and a randomized comparative study.
brexucabtagene autoleucel	MCL, ALL	ORR and DOR (MCL), Overall remission rate (CR/CRi) (ALL) ^b^	Whether observed responses are maintained over the long term and can be confirmed in broader patient populations.	Long-term evaluation of efficacy and safety through extended follow-up and prospective registry-based studies.
exagamglogene autotemcel	SCD/TDT	VF12 for SCD; TI12 for TDT ^a^	Whether VOC-free benefit in SCD and transfusion independence in TDT are maintained over the long term.	Long-term evaluation of efficacy and safety across TDT and SCD populations, including pediatric patients, extended follow-up and registry-based evidence collection.
betibeglogene autotemcel	TDT	Transfusion independence ^a^	Whether transfusion independence is maintained over the long term.	Long-term evaluation of efficacy and safety and extended follow-up.
nalotimagene carmaleucel	Adjunctive treatment in haploidentical HSCT	Overall survival ^a^	Whether the observed survival benefit can be confirmed.	Confirmation of efficacy and safety through a randomized phase III trial.

Remaining uncertainty at approval was descriptively characterized through an integrated review of approval-time efficacy evidence, EMA public assessment reports, and the specific obligations. The resulting summary describes the regulatory relationship between the unresolved efficacy question and the evidence expected after approval. Post-approval evidence required was summarized from specific obligations imposed at the time of conditional marketing authorization. a. Approval-time evidence representing a direct clinical outcome. b. Approval-time evidence representing a tumor response-based endpoint, including ORR, DOR, and CR/CRi. ALL = acute lymphoblastic leukemia; CR = complete remission; CRi = complete remission with incomplete hematologic recovery; DOR = duration of response; FVIII = factor VIII; MCL = mantle cell lymphoma; HSCT = hematopoietic stem cell transplantation; ORR = overall response rate; SCD = sickle cell disease; SMA = spinal muscular atrophy; TDT = transfusion-dependent β-thalassemia; TI12 = transfusion independence for at least 12 months; VF12 = freedom from severe vaso-occlusive crises for at least 12 months; VOC = vaso-occlusive crisis.

**Table 4:** Comparison with existing regulatory research on gene therapy.

Author	Published Year	Research Region	Research Topic	Reference
Vokinger KN, Glaus CEG, Kesselheim AS	2023	US, EU, Switzerland	Approved products, Indications, Therapeutic values	[25]
Jeong H, Purja S, Kim E	2024	US, EU, Japan, South Korea	Regulations, Guidelines and procedures of expedited programs, Approved products, Orphan drug designation	[5]
Delshad M, Davoodi-Moghaddam Z, Khademi M, et al.	2025	Worldwide	Status of current clinical trials, Indications	[16]
Ginn SL, Mandwie M, Alexander IE, et al.	2024	Worldwide	Geographical distribution of clinical trials, Indications, Vector Type, Transferred gene	[17]
Tandulje AA, Varpe PC, Chaugule PD, et al.	2025	US	Expedited regulatory pathways, Approval trends, Therapeutic area-specific trends	[24]
Hwang WYK, Bari S, Kawamata S, et al.	2025	Asia	Regulatory frameworks, Approved products, Pricing trends, Reimbursement policies	[26]
Iglesias-López C, Vallano A, Agustí A.	2026	US, EU	Approved products, Regulatory pathway, Quality issues	[9]
Our study	2026	US, Europe	Relationship among approval-time efficacy evidence, remaining uncertainty, and post-approval evidence requirements	–

EU = European Union, US = United States.

## Data Availability

The original contributions presented in this study are included in the article/Supplementary Material. Further inquiries can be directed to the corresponding authors.

## Supplementary Materials

The following supporting information can be downloaded at https://www.mdpi.com/article/10.3390/biotech15040082/s1: Supplementary Table S1: Original FDA Postmarketing Requirements for Gene Therapy Products Granted Accelerated Approval; Supplementary Table S2: Original EMA Specific Obligations for Gene Therapy Products Granted Conditional Marketing Authorisation; Supplementary Table S3: Frequency of Post-Approval Evidence Requirement Categories among Gene Therapy Products Granted FDA AA or EMA CMA.

## Abbreviations

The following abbreviations are used in this manuscript:

AA	Accelerated approval
AADC	Aromatic L-amino acid decarboxylase
AAV	Adeno-Associated Virus
ALL	Acute lymphoblastic leukemia
BCL-2	B-Cell Lymphoma 2
BLA	Biologics License Application
BTK	Bruton’s tyrosine kinase
CALD	Cerebral adrenoleukodystrophy
CAR-T	Chimeric antigen receptor-T cell
CLL	Chronic lymphocytic leukemia
CMA	Conditional marketing authorization
CR	Complete remission
CRi	Complete remission with incomplete hematologic recovery
DPd	Daratumumab, Pomalidomide, and Dexamethasone
DMD	Duchenne muscular dystrophy
DOR	Duration of response
EMA	European Medicines Agency
EPAR	European Public Assessment Report
FDA	Food and Drug Administration
FL	Follicular lymphoma
FVIII	Factor VIII
HLA	Human leukocyte antigen
HSCT	Hematopoietic stem cell transplantation
MAH	Marketing Authorisation Holder
MCL	Mantle cell lymphoma
MFD	Major Functional Disability
NCT	ClinicalTrials.gov identifier
NFS	Neurologic Function Score
NSAA	North Star Ambulatory Assessment
ORR	Overall Response Rate
OS	Overall Survival
PFS	Progression-Free Survival
PMR	Postmarketing requirement
PVd	Pomalidomide, Bortezomib, and Dexamethasone
SCD	Sickle cell disease
SMA	Spinal muscular atrophy
SO	Specific Obligation
SBRA	Summary Basis for Regulatory Action
TDT	Transfusion-dependent β-thalassemia
TI12	Transfusion Independence for at Least 12 months
VF12	Freedom from Severe Vaso-Occlusive Crises for at Least 12 Months
VOC	Vaso-occlusive crisis
