# Supplementary material for: Addressing Efficacy Uncertainties in Expedited Programs for Gene Therapy Products: A Comparative Assessment of Post-Approval Requirements by the FDA and EMA
Source: BioTech (Basel). 2026 Sep 25;15(4):82. doi: 10.3390/biotech15040082 (PMC13702514; doi:10.3390/biotech15040082)
Supplement: Supplementary file 1 [file biotech-15-00082-s001.zip › biotech-4549341-supplementary.pdf]

Table S1. Original FDA Postmarketing Requirements for Gene Therapy Products Granted Accelerated Approval

Pharmaceutical Name	Source document	Section	Original Postmarketing Requirement(s)
eladocagene exuparvovec	FDA SBRA (2024-11-13)	Section 11.c, PMR 1	Submit the clinical reports and datasets of clinical studies conducted in patients with AADC deficiency in the United States treated with KEBILIDI to verify and describe its clinical benefit. Such studies should, at minimum, evaluate the product's effects on the serious manifestations of AADC deficiency in adult and pediatric patients, including but not limited to motor function.
delandistrogene moxeparvovec (ambulatory patients)	FDA SBRA (2023-06-21)	Section 11.c, PMR 1	Completion of Study SRP-9001-301 Part 1, an ongoing, randomized, double-blinded clinical trial intended to describe and verify clinical benefit of ELEVIDYS in ambulatory patients with DMD. The study compares ELEVIDYS to placebo in 125 ambulatory patients with DMD with a confirmed mutation in the DMD gene. The primary endpoint is performance on the NSAA. The trial completion date is September 30, 2023.
delandistrogene moxeparvovec (non-ambulatory patients)	FDA PMR Database (BLA 125781, S-34)	Requirement No. 1	Conduct and submit the results of a randomized, controlled trial to verify and confirm the clinical benefit of delandistrogene moxeparvovec-rokl in patients with Duchenne's muscular dystrophy, who are non-ambulatory and have a confirmed mutation in the DMD gene.
afamitresgene autoleucel	FDA SBRA (2024-08-01)	Section 11.c, PMR 1	Submit the Final Clinical Study Report, including datasets from ADP-0044-002 Cohorts 2 and 3, to verify and describe the clinical benefit of afamitresgene autoleucel, through more precise estimation of the overall response rate and mature response duration per independent review assessment, in adults with unresectable or metastatic synovial sarcoma who have received prior chemotherapy, are HLA-A*02:01P, -A*02:02P, -A*02:03P, or -A*02:06P positive, and whose tumor expresses the MAGE-A4 tumor antigen as determined by FDA-approved or cleared companion diagnostic devices. Overall response rate and duration of response will be assessed by independent review and all patients will be followed for at least 15 months to assess duration of response.
lisocabtagene maraleucel	FDA PMR Database (BLA 125714, S-225)	Requirement No. 1	Collect and submit the final report, including datasets from the TRANSCEND FL clinical trial (NCT04245839) to verify and describe the clinical benefit of lisocabtagene maraleucel (BREYANZI) in adult patients with relapsed or refractory follicular lymphoma (FL) after two or more lines of systemic therapy (including an anti-CD20 antibody and an alkylating agent). All partial and complete responders should have completed at least 24 months of follow up starting from the initial objective response.
	FDA PMR Database (BLA 125714, S-205)	Requirement No. 1	Conduct a single arm study of lisocabtagene maraleucel in patients with relapsed or refractory chronic lymphocytic leukemia (CLL) or small lymphocytic lymphoma (SLL) who have received at least 2 prior lines of therapy, including a prior Bruton tyrosine kinase (BTK) inhibitor and a B-cell lymphoma 2 (BCL-2) inhibitor, to evaluate overall response rate and durability. The study must include a total of 50 treated patients and durable response should be based on a minimum follow-up of 15 months after first objective disease response.
tisagenlecleucel	FDA PMR Database (BLA 125646, S-663)	Requirement No. 1	Conduct a randomized phase 3 trial in adult patients with r/r FL. Patients will be randomized to tisagenlecleucel or an investigator's choice of regimens consistent with the standard of care. The primary endpoint will be PFS with secondary endpoints that include OS and ORR.
axicabtagene ciloleucel	FDA PMR Database (BLA 125643, S-248)	Requirement No. 1	A randomized phase 3 trial of axicabtagene ciloleucel in patients with relapsed or refractory FL. Patients will be randomized to axicabtagene ciloleucel or to an investigator's choice of regimens consistent with the standard of care. The primary endpoint will be PFS, with secondary endpoints that include ORR and OS.

Pharmaceutical Name	Source document	Section	Original Postmarketing Requirement(s)
brexucabtagene autoleucel	FDA Approval letter (2020-07-24)	Accelerated Approval Required Studies, No. 1	Complete additional follow-up of all 68 subjects treated with brexucabtagene autoleucel in ZUMA-2 Cohort 1 to a minimum of 18 months from the time of first response. Data will continue to be collected according to the ZUMA-2 protocol established schedule of assessments.
		Accelerated Approval Required Studies, No. 2	Conduct a study of brexucabtagene autoleucel treatment of subjects with relapsed or refractory mantle cell lymphoma who have not been exposed to a Bruton tyrosine kinase (BTK) inhibitor. A cohort of subjects naive to BTK inhibitor therapy will be added to the ongoing ZUMA-2 study to fulfill this requirement. Eighty-six subjects will be enrolled. The primary efficacy endpoint will be objective response rate with a supportive efficacy endpoint of duration of response based on a minimum follow-up of 18 months after first objective disease response.
elivaldogene autotemcel	FDA SBRA	Section 11.c, PMR 1	Follow all subjects who received elivaldogene autotemcel in Studies ALD-102 and ALD-104 to assess event-free survival (i.e., alive without Major Functional Disability (MFD) or need for hematopoietic stem cell transplant (HSCT)) for a minimum of ten years following administration of elivaldogene autotemcel.
		Section 11.c, PMR 2	Investigate event-free survival for at least five years post-treatment in 24 boys with more advanced early active, cerebral adrenoleukodystrophy (CALD) [(based on baseline Loes scores and Neurologic Function Score (NFS)] who will be newly treated with elivaldogene autotemcel (SKYSONA).

The table provides the source information used to derive the approval-time uncertainty and the summarized post-approval evidence requirements presented in Table 2. Where multiple FDA postmarketing requirements were imposed for a single product, they were reviewed collectively and consolidated into a product-level summary in Table 2.

Table S2. Original EMA Specific Obligations for Gene Therapy Products Granted Conditional Marketing Authorisation

Pharmaceutical Name	Source document	Section	Original Specific Obligation(s)
fidanacogene elaparvovec	EMA EPAR (EMA/323453/2024)	Specific Obligations to complete post-authorisation measures for the conditional marketing authorisation	In order to confirm the efficacy and safety of BEQVEZ in adults with severe and moderately severe haemophilia B (congenital factor IX deficiency) without a history of factor IX inhibitors and without detectable antibodies to variant AAV serotype Rh74, the MAH should submit interim results (6 years of data) of pivotal Study C0371002 with 45 subjects who received a dose calculated using actual batch concentration and at least 34-month data of patients who received a dose based on nominal concentration dosing.
			In order to confirm the efficacy and safety of BEQVEZ in adults with severe and moderately severe haemophilia B (congenital factor IX deficiency) without a history of factor IX inhibitors and without detectable antibodies to variant AAV serotype Rh74, the MAH should submit the final results (5 years of data) of long-term follow-up Study C0371003 with 14 subjects who received 5×10^{11} vector genomes per kg (vg/kg) of body weight.
etranacogene dezaparvovec	EMA EPAR (EMA/46569/2023)	Specific Obligations to complete post-authorisation measures for the conditional marketing authorisation	In order to confirm the efficacy and safety of etranacogene dezaparvovec in adult patients with severe and moderately severe Haemophilia B (congenital Factor IX deficiency) without a history of Factor IX inhibitors, the MAH should submit the final results (5 years of data) of pivotal Study CT-AMT-061-02 with 54 subjects.
			In order to confirm the efficacy and safety of etranacogene dezaparvovec in adult patients with severe and moderately severe Haemophilia B (congenital Factor IX deficiency) without a history of Factor IX inhibitors, the MAH should submit the final results including 5 years follow-up of the pivotal Study CT-AMT-061-01.
			In order to confirm the efficacy and safety of etranacogene dezaparvovec in adult patients with severe and moderately severe Haemophilia B (congenital Factor IX deficiency) without a history of Factor IX inhibitors, irrespective of baseline anti-AAV5 neutralising antibody titre, the MAH should submit the 1-year follow-up interim analysis report after the first 50 subjects are enrolled in Study CSL222_4001.
valoctocogene roxaparvovec	EMA EPAR (EMA/685615/2022)	Specific Obligations to complete post-authorisation measures for the conditional marketing authorisation	In order to confirm the efficacy and safety of Roctavian in adults with severe haemophilia A (congenital factor VIII deficiency) without a history of factor VIII inhibitors and without detectable antibodies to adeno-associated virus serotype 5 (AAV5), the MAH should submit the final results including 5 years follow-up of the phase 3, single arm study 270-301.
			In order to confirm the efficacy and safety of Roctavian, adequate corticosteroid regimen and to identify predictive factors for no or low response in adults with severe haemophilia A (congenital factor VIII deficiency), the MAH should submit the final results of the phase 3 single arm study 270-303 in patients receiving a prophylactic corticosteroid regimen. Interim data from open-label studies 270-203 and 270-205 should also be provided.
onasemnogene abeparvovec	EMA EPAR (EMA/200482/2020)	Specific Obligations to complete post-authorisation measures for the conditional marketing authorisation	In order to confirm the efficacy and safety and tolerability of a single dose of Zolgensma in patients younger than 6 months of age with Spinal Muscular Atrophy Type 1 with One or Two SMN2 Copies the MAH should submit final data on Study AVXS-101-CL-303-CL-303
			In order to confirm the efficacy and safety and tolerability of a single dose of Zolgensma in patients younger than 6 months of age with Spinal Muscular Atrophy Type 1 with One or Two SMN2 Copies the MAH should submit interim and final data on Study Zolgensma-CL-302
			In order to confirm the efficacy and safety and tolerability of a single dose of Zolgensma in patients to genetically diagnosed and pre-symptomatic patients equal or younger than 6 weeks of age at time of treatment with

Pharmaceutical Name	Source document	Section	Original Specific Obligation(s)
			SMA with bi-allelic deletion of SMN1 with 2 or 3 copies of SMN2, the MAH should submit interim and final data on Study Zolgensma-CL-304
obecabtagene autoleucel	EMA EPAR (EMA/197444/2025)	Specific Obligations to complete post-authorisation measures for the conditional marketing authorisation	In order to confirm the long-term efficacy and safety of Aucatzyl in adult patients with relapsed or refractory B cell precursor acute lymphoblastic leukaemia, the MAH shall submit final results of the FELIX clinical study, an open-label, single arm Phase Ib/II study of obecabtagene autoleucel in adult patients with relapsed or refractory B cell precursor acute lymphoblastic leukaemia.
			In order to confirm the efficacy and safety of Aucatzyl in adult patients with relapse or refractory B cell precursor acute lymphoblastic leukaemia, the MAH shall submit the results of a prospective, non-interventional study investigating efficacy and safety based on data from the same registry used to characterize the long-term safety and efficacy of Aucatzyl, according to an agreed protocol.
ciltacabtagene autoleucel	EMA EPAR (EMA/594558/2022)	Specific Obligations to complete post-authorisation measures for the conditional marketing authorisation	In order to confirm the efficacy and safety of Carvykti in adult patients with relapsed and refractory multiple myeloma, who have received at least three prior therapies, including a proteasome inhibitor, an immunomodulatory agent and an anti-CD38 antibody and have demonstrated disease progression on the last therapy, the MAH should submit the final study results of the pivotal study CARTITUDE-1 (MMY2001).
			In order to confirm the efficacy and safety of Carvykti in adult patients with relapsed and refractory multiple myeloma, who have received at least three prior therapies, including a proteasome inhibitor, an immunomodulatory agent and an anti-CD38 antibody and have demonstrated disease progression on the last therapy, the MAH should submit the results of the Phase 3 study CARTITUDE-4 (MMY3002) comparing the efficacy and safety of Carvykti vs. PVd or DPd in subjects with relapsed and lenalidomide-refractory multiple myeloma.
idecabtagene vicleucel	EMA EPAR (EMA/409800/2021)	Specific Obligations to complete post-authorisation measures for the conditional marketing authorisation	In order to confirm the efficacy and safety of Abecma in adult patients with relapsed and refractory multiple myeloma who have received at least three prior therapies, the MAH should submit 24 months post-Abecma infusion follow-up data (in the enrolled and treated population) of the pivotal study KarMMa (MM-001).
			In order to confirm the efficacy and safety of Abecma in adult patients with relapsed and refractory multiple myeloma who have received at least three prior therapies, the MAH should submit the results of the Phase 3 study KarMMa-3 (MM-003) comparing the efficacy and safety of Abecma vs. standard triplet regimens in subjects with relapsed and refractory multiple myeloma.
brexucabtagene autoleucel (Mantle cell lymphoma)	EMA EPAR (EMA/588798/2020)	Specific Obligations to complete post-authorisation measures for the conditional marketing authorisation	In order to confirm the long-term efficacy and safety of Tecartus in adult patients with relapsed or refractory MCL and the Benefit/Risk balance in the female, elderly and severely diseased patients, the MAH shall submit the results of a prospective study investigating efficacy and safety based on data from the same registry used to characterise the long-term efficacy and safety of Tecartus, according to an agreed protocol.
			In order to confirm the long-term efficacy and safety of Tecartus in adult patients with relapsed or refractory MCL the MAH shall submit the 24 months follow-up data from all treated patients in cohort 1 of the pivotal study ZUMA-2.
brexucabtagene autoleucel	EMA EPAR (EMA/683619/2022)	Specific Obligations to complete post-	In order to confirm the long-term efficacy and safety of Tecartus in adult patients with r/r ALL, the MAH shall submit follow-up results of the ZUMA-3 clinical study (Part 1 and Part 2).

Pharmaceutical Name	Source document	Section	Original Specific Obligation(s)
(Acute lympho-blastic leuke-mia)		authorisation measures for the conditional mar-keting authorisation	In order to confirm the long-term efficacy and safety of Tecartus in adult patients with r/r ALL, the MAH should conduct and submit the results of a prospective, observational study based on data from a registry, ac-cording to an agreed protocol.
exagamglogene autotemcel	EMA EPAR (EMA/6332/2024)	Specific Obligations to complete post-authori-sation measures for the conditional marketing authorisation	In order to confirm the efficacy and safety of exagamglogene autotemcel in patients with transfusion-dependent β-thalassemia (TDT) aged 12 years and older, the MAH should submit the final results from the study 111, a phase 1/2/3 study evaluating the safety and efficacy of a single dose of exagamglogene autotemcel in subjects with transfusion-dependent β-thalassemia.
			In order to confirm the efficacy and safety of exagamglogene autotemcel in patients with severe sickle cell dis-ease (SCD) aged 12 years and older, the MAH should submit the final results from the study 121, a Phase 1/2/3 Study to evaluate the safety and efficacy of a single dose of exagamglogene autotemcel in subjects with severe SCD.
			In order to confirm the efficacy and safety of exagamglogene autotemcel in patients with severe sickle cell dis-ease (SCD) aged 12 years and older, the MAH should submit the final results from the study 151, a Phase 3 Study to evaluate the safety and efficacy of a single dose of exagamglogene autotemcel in paediatric patients with severe SCD aged between 2 to 11 years.
			In order to confirm the efficacy and safety of exagamglogene autotemcel in patients with transfusion-dependent β-thalassemia (TDT) and severe sickle cell disease (SCD) aged 12 years and older, the MAH should submit the interim results from the study 161, a phase 3b study to evaluate the efficacy and safety of a single dose of ex-agamglogene autotemcel in subjects with TDT or severe SCD.
			In order to confirm the efficacy and safety of exagamglogene autotemcel in patients with severe sickle cell dis-ease (SCD) aged 12 years and older, the MAH should submit the final results from the study 171, a phase 3 study to evaluate the safety and efficacy of a single dose of exagamglogene autotemcel in subjects with severe SCD, βS/βC genotype.
			In order to confirm the efficacy and safety of exagamglogene autotemcel in patients with transfusion-dependent β-thalassemia (TDT) and severe sickle cell disease (SCD) aged 12 years and older, the MAH should conduct and submit the interim results of a study based on data from a registry, according to an agreed protocol. Yearly pro-gress reports shall also be submitted.
			In order to confirm the efficacy and safety of exagamglogene autotemcel in patients with transfusion-dependent β-thalassemia (TDT) and severe sickle cell disease (SCD) in patients aged 12 years and older, the MAH should submit the interim results from study 131, a long term follow-up open-label trial evaluating the safety and effi-cacy of exagamglogene autotemcel for 15 years in subjects with TDT and severe SCD who received treatment with exagamglogene autotemcel in previous clinical trials.
betibeglogene autotemcel	EMA EPAR (EMA/56140/2020/ Corr.1)	Specific Obligations to complete post-authori-sation measures for the conditional marketing authorisation	In order to confirm the efficacy and safety of Zynteglo in patients 12 years and older with transfusion-depend-ent β-thalassaemia (TDT) who do not have a β0/β0 genotype, the MAH should submit interim and final data on Study HGB-207.
			In order to confirm the efficacy and safety of Zynteglo in patients 12 years and older with transfusion-depend-ent β-thalassaemia (TDT) who do not have a β0/β0 genotype, the MAH should submit interim and final data

Pharmaceutical Name	Source document	Section	Original Specific Obligation(s)
			from patients with a severe non-β0/β0 genotype such as IVS-I-110/IVS-I-110 and IVS-I-110/β0 from Study HGB-212.
			In order to confirm the efficacy and safety of Zynteglo in patients 12 years and older with transfusion-dependent β-thalassaemia (TDT) who do not have a β0/β0 genotype, the MAH should submit interim data and the 5 years follow-up results of Study LTF-303.
nalotimagene carmaleucel	EMA EPAR (EMA/CHMP/589978/2016)	Specific Obligations to complete post-authorisation measures for the conditional marketing authorisation	The MAH shall complete, within the stated timeframe, the below measures: In order to confirm the efficacy and safety of Zalmoxis as an adjunctive treatment in haploidentical haematopoietic stem-cell transplantation of adult patients with high-risk haematological malignancies, the MAH should submit the results of study TK008, a randomized phase III trial of haploidentical HCT with an add back strategy of HSV-Tk donor lymphocytes in patients with high risk acute leukaemia. The clinical study report should be submitted by March 2021.

The table provides the source information used to derive the summarized post-approval evidence requirements presented in Table 3. Where multiple specific obligations were imposed for a single product, they were reviewed collectively and consolidated into a product-level summary in Table 3.

Table S3. Frequency of Post-Approval Evidence Requirement Categories among Gene Therapy Products Granted FDA AA or EMA CMA

Post-approval evidence requirement category	FDA AA, n = 8, n (%)	EMA CMA, n = 11, n (%)
Extended follow-up or maturation of outcomes	4 (50.0%)	10 (90.9%)
Randomized comparative studies	3 (37.5%)	3 (27.3%)
Registry-based evidence collection	0 (0%)	3 (27.3%)
Additional cohort or patient population evidence	2 (25.0%)	2 (18.2%)

Note: A single product could be included in more than one category if multiple types of post-approval evidence requirements were identified. Therefore, the same product may be counted in multiple categories. Each product was counted once within each applicable category. The unit of analysis was the product, consistent with Tables 2 and 3. Categories were assigned from the product-level summaries of post-approval evidence requirements presented in Tables 2 and 3.

Classification detail: FDA AA: extended follow-up or maturation of outcomes, afamitresgene autoleucel, lisocabtagene maraleucel, brexucabtagene autoleucel, and elivaldogene autotemcel; randomized comparative studies, delandistrogene moxeparvovec, tisagenlecleucel, and axicabtagene ciloleucel; registry-based evidence collection, none; additional cohort or patient population evidence, lisocabtagene maraleucel and brexucabtagene autoleucel. EMA CMA: extended follow-up or maturation of outcomes, 10 of 11 products (all except nalotimagene carmaleucel); randomized comparative studies, ciltacabtagene autoleucel, idecabtagene vicleucel, and nalotimagene carmaleucel; registry-based evidence collection, obecabtagene autoleucel, brexucabtagene autoleucel, and exagamglogene autotemcel; additional cohort or patient population evidence, onasemnogene abeparvovec and exagamglogene autotemcel.
